# A unified single-cell atlas of HNSCC: Toward characterizing HPV- and sex-associated TME variability

**DOI:** 10.1016/j.isci.2026.115863

**Published:** 2026-04-22

**Authors:** Cristina Conde-Lopez, Divyasree Marripati, Maria Jose Besso, Mareike Roscher, Rui Han, Wahyu Wijaya Hadiwikarta, Moshe Elkabets, Jochen Hess, Ina Kurth

**Affiliations:** 1 German Cancer Research Center Heidelberg (DKFZ), Radiooncology/Radiobiology, Heidelberg, Germany; 2 Department of Otorhinolaryngology, Head and Neck Surgery, Heidelberg University Hospital, Heidelberg, Germany; 3 The Shraga Segal Department of Microbiology, Immunology, and Genetics, Ben-Gurion University of the Negev, Beer-Sheva, Israel; 4 Faculty of Health Sciences, Ben-Gurion University of the Negev, Beer-Sheva, Israel; 5 Department of Otorhinolaryngology, Head and Neck Tumors, Heidelberg University Hospital, 69120 Heidelberg, Germany; 6 German Cancer Research Center Heidelberg (DKFZ), Service Unit for Radiopharmaceuticals and Preclinical Studies, Heidelberg, Germany; 7 German Cancer Consortium (DKTK), Core Center Heidelberg, 69120 Heidelberg, Germany

**Keywords:** bioinformatics, biological database, computational bioinformatics, cancer

## Abstract

Head and neck squamous cell carcinoma (HNSCC) is highly heterogeneous, with variations driven by human papillomavirus (HPV) status and sex. However, existing single-cell RNA sequencing (scRNA-seq) studies are often limited in sample size and lack standardized methodologies, limiting cross-study comparisons. To address this, we integrated scRNA-seq data from 78 patients (274,911 cells) across multiple studies, creating a unified HNSCC atlas that harmonizes annotations and enables robust tumor microenvironment (TME) analyses. Using STACAS for semi-supervised integration and automated annotation tools such as Ikarus and scGate, we improved tumor and immune cell classification. Leveraging our atlas, we identified HPV-specific shifts in immune and stromal composition, with HPV+ tumors enriched in adaptive immune cells and HPV− tumors showing more stromal and myeloid populations. Preliminary sex-stratified analyses suggested distinct microenvironmental patterns, warranting further investigation. This publicly available atlas provides a comprehensive framework for reproducibly studying HNSCC biology, improving patient stratification, and may help inform personalized therapies.

## Highlights

• Unified single-cell atlas integrates 78 patients with HNSCC across four studies
• STACAS, scGate, and Ikarus improved harmonized tumor cell annotation
• Public HNSCatlas resource supports reproducible cross-cohort analyses
• HPV status shaped immune and stromal composition in the HNSCC atlas

## Graphical abstract

**Figure:**
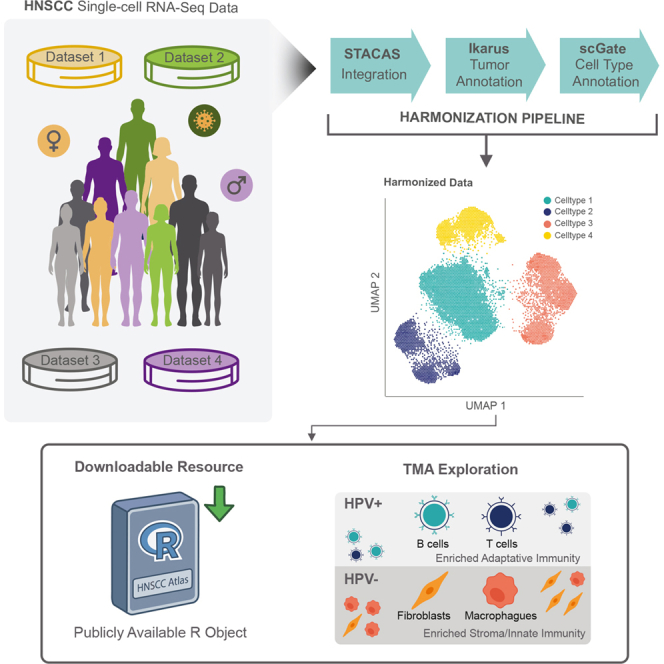

## Introduction

Head and neck squamous cell carcinoma (HNSCC) accounts for over 90% of head and neck cancers (HNCs), with human papillomavirus (HPV) infection as a major etiological risk factor, particularly in oropharyngeal cancers.^1^^,^^2^^,^^3^ HNSCC also exhibits significant sex differences, with a higher incidence in males, traditionally linked to tobacco and alcohol use, though recent evidence suggests additional biological and molecular contributors.^4^^,^^5^ Despite advances in treatment, the five-year overall survival (OS) remains below 50%, and relapse rates exceed 50%, indicating the need for improved therapeutic strategies.^1^ Managing HNSCC is particularly challenging due to high intra- and inter-tumor heterogeneity, yet current treatment approaches remain largely uniform, highlighting the necessity to improve outcomes through better patient stratification.^6^^,^^7^

For the past decade, transcriptomics has largely relied on bulk RNA sequencing, which captures aggregate gene expression but obscures cellular heterogeneity. While collaborative initiatives such as The Cancer Genome Atlas (TCGA) have identified tumor subtypes and molecular signatures, bulk RNA sequencing cannot resolve the distinct contributions of different cell types, limiting its ability to reveal key therapeutic targets.^8^^,^^9^^,^^10^^,^^11^^,^^12^^,^^13^ Single-cell RNA sequencing (scRNA-seq) has transformed cancer research by enabling high-resolution analysis of the tumor microenvironment (TME).^14^ This approach uncovers interactions between tumor, immune, and stromal cells, which are critical for cancer progression and treatment response.^15^ However, inconsistencies in sample sizes, annotation standards, and methodologies across studies complicate the unified understanding of HNSCC through scRNA-seq. For example, variations in chromosomal aberrations, HPV gene expression, and macrophage polarity have been identified across studies, yet differences in focus and specifically cell annotations have resulted in fragmented knowledge, emphasizing the need for a standardized framework.^2^^,^^16^

HNSCC research faces several challenges, including high intra- and inter-patient TME heterogeneity driven largely by tumor cells and the stromal component. Another major hurdle is the inconsistency in tumor cell annotation, as classification criteria vary based on gene expression and copy number variation (CNV) patterns. The lack of standardization across studies affects data comparability and impedes progress in targeted therapy development. Machine learning-based approaches offer potential solutions for improving tumor cell annotation consistency, while the integration of multi-study datasets helps address limitations of small sample sizes.^14^^,^^17^^,^^18^ Additionally, the dynamic nature of cells presents a persistent problem, as scRNA-seq captures only snapshots of cellular states, complicating comparability across studies.^19^ Advances in AI-driven annotation tools and community-led initiatives, such as “single-cell best practices” and “scRNA-tools,” aim to establish standardized workflows and improve data harmonization.^20^^,^^21^ Resource-intensive single-cell techniques remain a challenge, but public datasets and computational strategies such as pseudobulking offer cost-effective alternatives to enhance uniform data reusability.

In this study, we present our newly developed pipeline for integrating multiple single-cell datasets to create a comprehensive atlas of HNSCC, with the aim of overcoming the limitations of isolated studies. By consolidating data from various independent studies into a unified “universal atlas,” we achieve a holistic view of the TME. The incorporation of key variables such as HPV status and sex enables systematic exploration of biological and molecular mechanisms that drive etiological factors and their impact on tumor behavior and patient outcomes. This atlas provides a high-resolution map of cellular diversity and functional interactions within the TME, bridging gaps in existing research and enhancing our understanding of cancer progression and treatment resistance. The pipeline and atlas are publicly available (https://github.com/DKFZ-E220/HNSCatlas) as a resource for the research community to facilitate further investigation and discovery.

## Results

### Generation and validation of the HNSCC atlas

**Figure 1:**
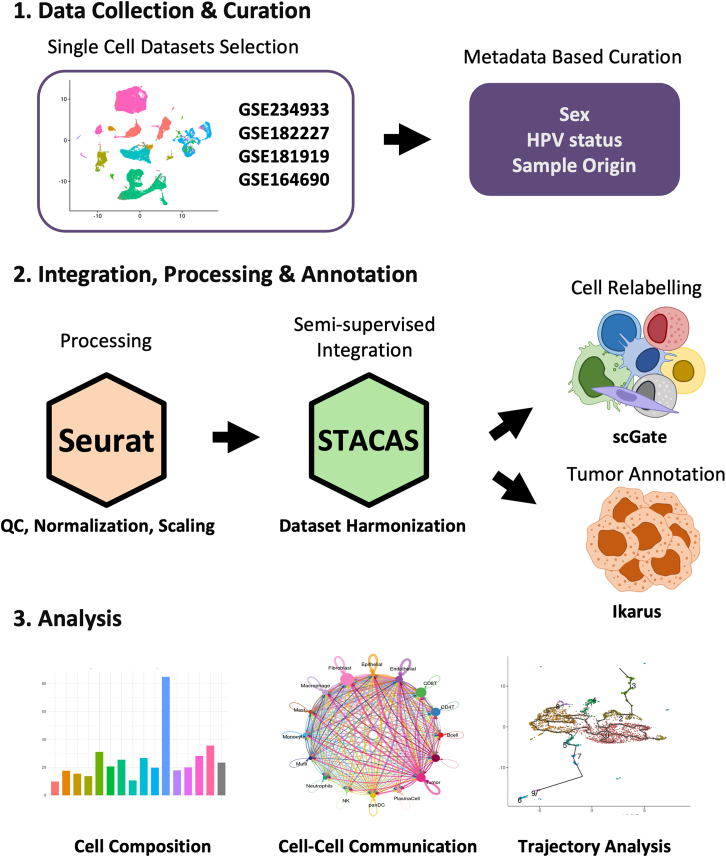
Workflow for creating the HNSCC atlas The workflow involves three main stages. First, single-cell RNA-seq datasets are collected and curated based on key metadata, including sex, HPV status, and sample origin. Next, data processing is performed with Seurat for quality control, normalization, and scaling, followed by dataset harmonization using STACAS for semi-supervised integration. Cell relabeling is refined with scGate, and tumor cells are specifically annotated using Ikarus. The harmonized atlas enables a range of downstream analyses, some of which are demonstrated in this study, while others represent potential applications of the resource.

To create a comprehensive HNSCC atlas, we built upon a previously published systematic overview of publicly available human HNSCC single-cell RNA-sequencing datasets.^14^ Based on this landscape, all publicly available datasets were systematically screened for inclusion (Table S1). Datasets were evaluated according to the availability of key clinical annotations (sex and HPV status), data modality, sample composition, and compatibility with integrative atlas construction. Datasets restricted to specific immune or stromal subsets, lacking essential clinical metadata, or representing distinct anatomical subtypes were excluded to ensure cross-cohort comparability. Following this evaluation, four publicly available scRNA-seq datasets were selected (GEO: GSE234933, GEO: GSE182227, GEO: GSE164690, and GEO: GSE181919). These datasets were chosen because they provide detailed and consistent clinical metadata, including sex, HPV status, and sample origin (normal tissue, primary tumor, or metastasis), enabling harmonized integration and stratified downstream analyses. This approach integrated data from 78 patients, comprising 274,911 cells after quality control filtering, significantly expanding beyond the typical range of 11–20 patients in individual HNSCC datasets (Table S2). Important data curation steps, such as removing low-quality data points and harmonizing variables, ensured a consistent data structure across studies, providing a strong foundation for integration. These initial steps of quality control, normalization, and scaling were performed using the Seurat pipeline. A schematic view of the atlas creation process and representative downstream applications is shown in Figure 1, while some analyses are demonstrated in this study, others illustrate potential uses of the resource.

**Figure 2:**
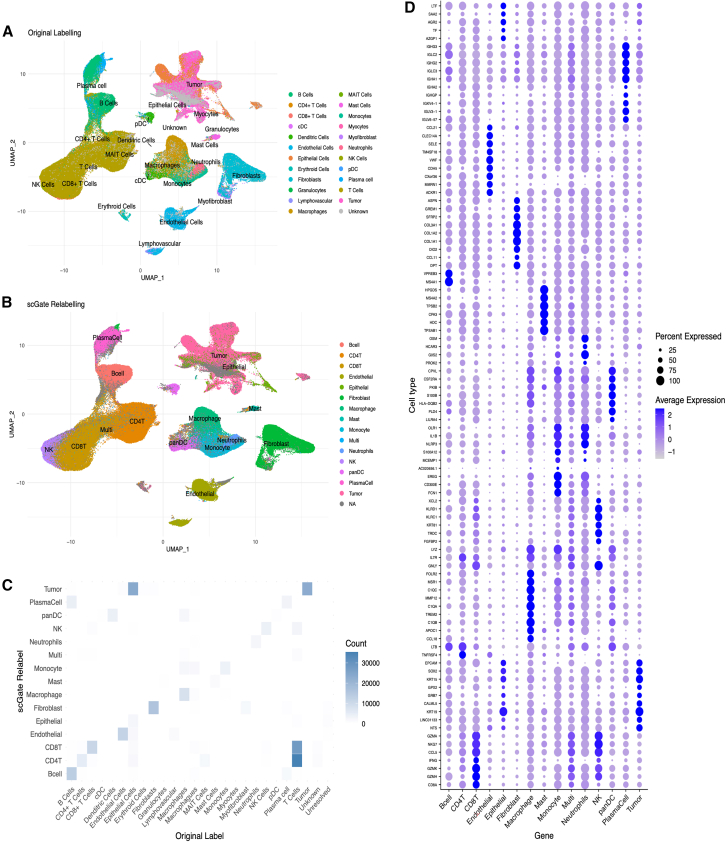
Integration and cell relabeling in the HNSCC atlas using scGate (A) UMAP visualization of the original cell type labels across the integrated single-cell RNA-seq datasets. (B) UMAP shows the updated cell annotations following scGate-based relabeling, with an additional tumor cell population annotated using the Ikarus classifier. This relabeling enhanced the resolution of tumor and non-tumor cells, particularly by reclassifying epithelial cells as tumor cells where appropriate. (C) Mosaic plot compares the distribution of original and scGate-relabeled cell types. The plot illustrates the reassignment of cells, especially the transition of epithelial cells to the tumor cell category. (D) Dot plot shows top differentially expressed genes across annotated cell types. This analysis highlights the most enriched markers in each major cell population.

The harmonization process significantly improved data compatibility across the HNSCC single-cell datasets, as shown in the Uniform Manifold Approximation and Projection (UMAP) plots (Figures 2A and 2B). The integration quality was quantitatively validated using established metrics such as LISI and ASW, confirming superior performance of the STACAS pipeline compared to alternative integration methods, including Harmony and Seurat CCA (Figure S1). This refinement led to a more cohesive clustering of cells, with increased density within each cell type and improved separation between distinct populations. While the number of annotated cell types decreased from 24 to 16, this reduction reflects the consolidation of overlapping or partially inconsistent annotations across studies, resulting in a more consistent and interpretable cell-type framework. The harmonization process improved cross-study comparability by standardizing original annotations to a common reference (Figure 2A), while scGate and Ikarus enabled further refinement of cell identities in a reproducible manner (Figure 2B). This was particularly relevant for tumor cells, which were underrepresented in the original labels. The application of Ikarus, a model-based classifier, expanded the annotated tumor cell population from 2,300 to nearly 50,000 cells, most of which clustered within epithelial regions of the UMAP. These improvements are also reflected in the mosaic plot (Figure 2C), where a clear shift from epithelial to tumor identity is observed in the relabeled dataset.

#### Automated annotation of cell types

TME cell types were annotated using automated gating models from scGate, which applies signature-based scoring to assign cells to predefined populations. This relabeling process refined the original annotations by resolving inconsistencies and enhancing cell-type resolution across samples. The UMAP plots (Figures 2A and 2B) illustrate the harmonization and increased consistency of label resolution across the integrated atlas, particularly for challenging populations such as T cell subtypes and fibroblasts.^22^^,^^23^ To evaluate the global effect of the relabeling process, the original and updated annotations were compared using a mosaic plot (Figure 2C), highlighting key transitions such as the separation of overlapping plasma and B cells. The T cell compartment, originally annotated as a single cluster, is now split into CD4 and CD8 subsets, reflecting increased resolution and consistency in immune classification across studies. To quantitatively assess the extent and consistency of these refinements, the Adjusted Rand Index (ARI = 0.338) and normalized mutual information (NMI = 0.516) between the original and relabeled annotations were computed. These values indicate moderate agreement, consistent with biologically meaningful restructuring rather than simple label preservation and support the improved coherence of the final annotations. Notably, multiple datasets originally used heterogeneous naming conventions for similar populations (e.g., plasma/B cell overlap or broad T cell labels), which were harmonized into consistent identities, thereby reducing cross-study annotation inconsistencies. To validate the final annotations, differential expression analysis was performed across all major cell types, identifying top markers such as CD8A, GZMH, and GZMK in CD8^+^ T cells, LTB and TNFRSF4 in CD4^+^ T cells, and ASPN, GREM1, SFRP2, COL3A1, and COL1A2 in fibroblasts (Figure 2D). Signature scoring further confirmed the selective enrichment of these gene sets within the expected populations (Figure S2), supporting the biological specificity and robustness of the automated annotation.

To further validate the pipeline’s applicability beyond HNSCC, the annotation framework was applied to an independent esophageal squamous cell carcinoma (ESCC) dataset.^24^ scGate and Ikarus successfully recovered major TME cell populations, including tumor, immune, and stromal compartments, with cell type assignments broadly consistent with the original labels (Figure S3). These results suggest the pipeline generalizes to other squamous cell carcinoma contexts with minimal adaptation.

#### inferCNV analysis for tumor cell reclassification

**Figure 3:**
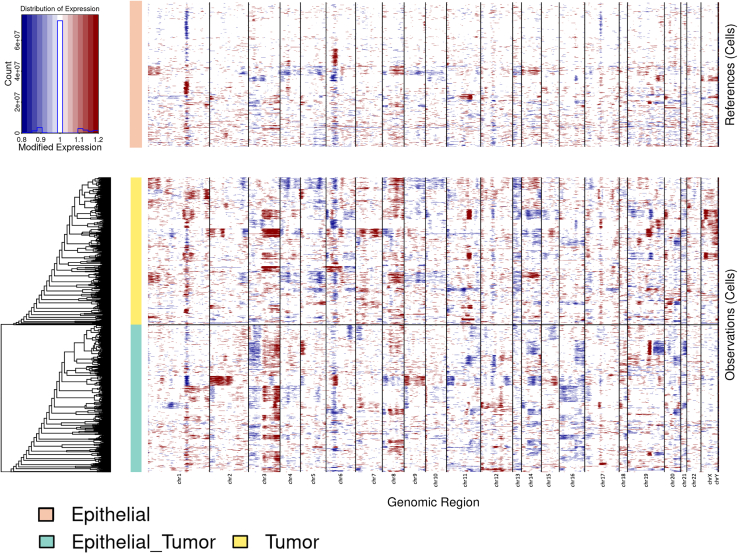
Validation of tumor cell reclassification using inferCNV analysis The copy number variation (CNV) analysis was performed on epithelial cells, originally annotated tumor cells, and cells reclassified from epithelial to tumor. Heatmaps show chromosomal instability across genomic regions, with red and blue representing gains and losses, respectively. Cells reclassified as tumor using the Ikarus algorithm displayed a higher level of chromosomal instability compared to epithelial cells, validating their tumor identity. This approach demonstrates the utility of Ikarus for identifying tumor cells based on gene expression-derived CNVs.

Since tumor cells typically exhibit higher chromosomal instability compared to normal epithelial cells, chromosomal CNV analysis can serve as an independent validation of transcription-based classification methods. Although reclassified tumor cells showed strong tumor-like expression signatures (Figure S2) and were confidently identified by the Ikarus algorithm, we further validated their classification using CNV analysis. We analyzed three groups: (1) originally annotated tumor cells, (2) epithelial cells, and (3) cells reclassified from epithelial to tumor using the Ikarus algorithm. While canonical HNSCC CNV events can vary across datasets and tumor subtypes, cells reclassified as tumor consistently showed elevated chromosomal instability relative to epithelial cells (Figure 3). This approach validated the utility of the Ikarus method, as it enables the identification of cells with significant CNV based on gene expression. In addition to CNV-based validation, tumor cells showed strong enrichment of epithelial/tumor-associated transcriptional signatures (Figure S2), further supporting their malignant identity.

#### Refinement of immune compartment annotations

Using gating models from scGate, the classification of immune cells was refined and deepened. Predefined subtype signatures from frameworks such as ProjectTILS were used to label key types such as CD4 and CD8 T cells and dendritic cells, ensuring consistent and reliable annotations across our dataset. To further validate the annotation of T cell subtypes, differential expression analysis was performed to identify top markers for each cluster, which showed strong concordance with expected gene expression patterns (Figure S4). A complete list of cell subtype abbreviations used in this study is provided in Table S8.

### Deep TME analysis across datasets using the unified HNSCC atlas

Next, the unified atlas was used to conduct an in-depth TME analysis of the 78 patients with HNSCC, overcoming previous limitations imposed by the small sample sizes of individual datasets and inconsistencies in cell annotation. The cohort included 46 HPV- and 32 HPV+ tumors, with a strong male predominance (62 males, 16 females), which limited the statistical power of some comparisons. Notably, within the HPV+ subgroup, only 2 samples were derived from female patients, precluding statistically meaningful sex-stratified comparisons in this context. Sex-stratified analyses should therefore be interpreted as exploratory and hypothesis-generating rather than definitive.

**Figure 4:**
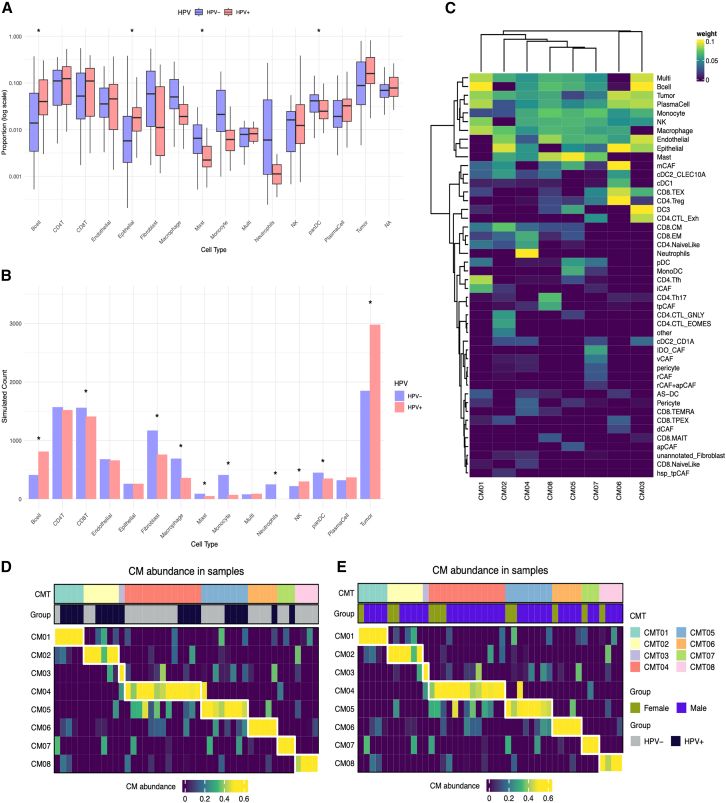
HPV-specific cell type distributions and higher-order coordination patterns in the HNSCC TME (A) Boxplots present per-patient cell type proportions in HPV-positive (HPV+) and HPV-negative (HPV-) tumors. Proportions are displayed on a log10 scale to improve the visualization of low-abundance populations. Statistical comparisons were performed using the Wilcoxon test, and significance is indicated as *p* < 0.05 (∗). Nominal *p*-values (Wilcoxon test) are shown; differences did not remain significant after correction for multiple testing. (B) Barplots show projected cell counts based on proportions for each cell type in HPV+ and HPV- tumors. Statistical comparisons were performed using Fisher’s exact test, and significance is indicated as *p* < 0.05 (∗). (C) Heatmap of CoVarNet-derived module weights across cell types, showing the contribution of each cell type to the eight identified co-variation modules (CM01–CM08). (D) Module abundance per patient stratified by HPV status. (E) Module abundance per patient stratified by sex.

To assess differences in cell type composition between HPV+ and HPV− tumors, per-patient proportions (excluding the CD45-sorted dataset GEO: GSE164690) were computed by dividing the number of cells of each type by the total number of cells per patient (Figure 4A), capturing interindividual variability in tumor composition. To complement this, cell type proportions were scaled to a fixed total (Figure 4B), generating simulated counts that provided a more standardized measure of relative abundance. While simulated counts reduced interpatient variation, patient-level proportions offered valuable context for individual heterogeneity. Both approaches revealed consistent trends with HPV+ tumors showing enrichment in adaptive immune cells, including B cells, plasma cells, and CD4/CD8 T cells, suggestive of a more active immune microenvironment, whereas HPV- tumors showed elevated levels of innate and stromal populations such as macrophages, monocytes, fibroblasts, and neutrophils, aligning with a more suppressive TME.^25^ Although these differences did not remain statistically significant after multiple testing correction across cell types, the directionality of the observed patterns was consistent across analytical approaches. NK cells were also more abundant in HPV+ tumors. To further explore TME heterogeneity, cell composition was stratified by both HPV status and patient sex (Figure S5; Tables S3–S7), revealing sex-specific differences in fibroblast and tumor cell abundance as well as varying levels of exhausted versus cytotoxic CD8 T cells, particularly in HPV+ tumors.

To investigate coordinated shifts in the TME, CoVarNet was applied, a computational framework that identifies cellular co-variation modules (CMs) by analyzing co-fluctuations in cell type abundances across tumors. This analysis yielded eight distinct co-variation modules (CM01–CM08), each composed of cell types that tend to increase or decrease together across patient samples (Figure 4C). The number of modules was determined based on the default hierarchical clustering parameters of CoVarNet and the stability of module composition across patients. For example, CM01 was enriched for CD4 Tfh cells, B cells, plasma cells, macrophages, and notably iCAFs, indicative of a humoral and immunoregulatory microenvironment. CM04 was dominated by neutrophils and CD8 EM cells, pointing to a more inflammatory composition. CM06, on the other hand, integrated CD4 Tregs, mCAFs, and CD8 TEX, potentially reflecting an exhausted or immunosuppressive microenvironmental niche.

The distribution of module abundance across tumors showed that CM01, CM02, and CM03 were more prominent in HPV+ tumors, whereas CM04, CM06, CM07, and CM08 were more abundant in HPV- tumors, suggesting that HPV- tumors may harbor more immunosuppressive or inflammatory TMEs, while HPV+ tumors are characterized by more coordinated lymphoid and stromal ecosystems (Figure 4D). When stratified by sex (Figure 4E), no clear pattern emerged; all modules were represented in both male and female tumors, with no strong enrichment apart from CM03, which was present in only a single male sample and is therefore not interpretable.

**Figure 5:**
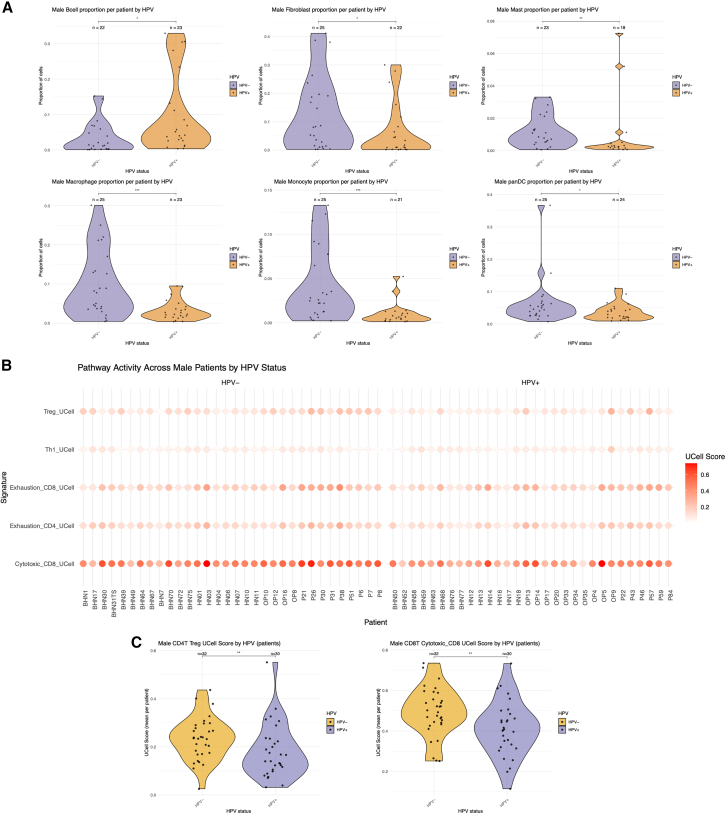
HPV status shapes immune cell composition and functional states in male HNSCC tumors (A) Violin plots show the relative proportions of major cell types across HPV+ and HPV− male patients. Significant differences were determined by the Wilcoxon rank-sum test (*p* < 0.05 (∗)). (B) Dotplot displays per-patient UCell scores for selected CD4^+^ and CD8^+^ T cell gene signatures (Treg, Th1, exhaustion, and cytotoxicity), stratified by HPV status. (C) Violin plots of Treg and cytotoxic CD8 T cell signature scores, which showed statistically significant differences between HPV+ and HPV- groups (Wilcoxon test; *p* < 0.05 (∗)).

Building on the coordinated ecosystem patterns identified above, the analysis next focused on male patients to explore differences in cell composition and immune activity between HPV+ and HPV- tumors. We restricted this analysis to males (*n* = 62; 32 HPV− and 30 HPV+) due to the limited number of female samples, which did not allow for a balanced comparison. Per-patient cell type proportions (excluding the CD45-sorted dataset GSE164690) revealed compositional differences between groups (Figure 5A). HPV+ tumors showed a trend toward increased B cell abundance, consistent with prior reports of enhanced adaptive immune infiltration in virally driven tumors, whereas HPV- tumors were enriched in innate immune populations, including monocytes, macrophages, and mast cells. After Benjamini-Hochberg correction, macrophages, monocytes, and mast cells remained significantly enriched in HPV− tumors, while differences in other populations, including B cells, did not remain significant after adjustment. To further dissect immune cell states, we evaluated pathway-level activity in CD4 and CD8 T cells using UCell scoring. Although scores varied across patients (Figure 5B), both the cytotoxic CD8 T cell and Treg signatures were significantly elevated in HPV- tumors (Figure 5C), suggesting a coexistence of cytotoxic and immunosuppressive programs within the same tumor context, a pattern that contrasts with previous reports of enhanced cytotoxicity in HPV+ tumors. This discrepancy may reflect differences in cohort composition, sample processing, or tumor-intrinsic heterogeneity and warrants further investigation.

## Discussion and outlook

The creation of a comprehensive single-cell atlas marks a significant advancement in the study of HNSCC. By integrating data from 78 patients and 274,911 cells across diverse clinical backgrounds, we have created a high-resolution map of the TME. This atlas represents an important step toward addressing dataset heterogeneity, offering a standardized framework for systematically exploring cellular composition and function across cohorts. This approach not only enhances the resolution of cellular phenotypes and facilitates the exploration of inter-dataset variability but also allows for the robust validation of findings across diverse patient samples and experimental conditions.

Although sex represents an important biological variable in HNSCC, the present atlas is limited by an imbalance in sex representation, particularly within HPV+ tumors. Consequently, sex-stratified analyses presented in this study should be interpreted as hypothesis-generating rather than definitive. Addressing sex-specific TME programs will require future studies with more balanced cohorts to enable adequately powered comparisons.

Compared to previous efforts in integrating single-cell data from HNSCC samples, such as the work by Dai et al., the approach stands out for incorporating an integration strategy and refined annotation methodologies that build upon existing approaches.^26^ While Dai et al. integrated five datasets using the standard Seurat pipeline with manual annotation based on cluster markers, we expanded upon this by incorporating STACAS for semi-supervised integration, leveraging existing labels to guide the harmonization process. In addition, we implemented automated annotation pipelines, including the Ikarus algorithm and scGate to ensure consistency and reproducibility across datasets. The reannotation of some epithelial cells as malignant was validated using inferCNV, which confirmed higher CNV counts, indicative of their tumorigenic nature.

We have generated a comprehensive single-cell atlas that integrates and harmonizes diverse scRNA-seq datasets from multiple HNSCC cohorts under unified annotation criteria. By combining semi-supervised integration with automated and reproducible annotation frameworks and distributing the result as a publicly accessible R package, this work provides not only a biological analysis but also a reusable resource designed to facilitate transparent cross-study comparison and future expansion. This unified framework enables holistic and stratified analysis of the TME, overcoming prior limitations imposed by small sample sizes and inconsistent cell annotations across individual studies. By incorporating key clinical variables such as HPV status and sex, our atlas allows for a detailed dissection of cellular compositions and immune states, which may help inform future studies on therapy response. The in-depth analysis of 78 tumors (46 HPV−, 32 HPV+), with a predominant representation of male patients (62 males, 16 females), points toward distinct differences in TME composition between HPV groups. HPV+ tumors tended to be enriched in adaptive immune components such as B cells and T cells, whereas HPV- tumors exhibited higher levels of stromal and innate immune populations, including macrophages, monocytes, fibroblasts, and mast cells. These patterns suggest that HPV- tumors harbor a more inflammatory and stromal-rich microenvironment, potentially associated with immune suppression. Our observations are further supported by recent studies suggesting a more robust immune activation in HPV+ cases.^27^ To capture higher-order relationships, CoVarNet was applied and allowed for the identification of distinct cellular co-variation modules reflecting coordinated immune and stromal ecosystems differentially abundant in HPV+ and HPV- tumors.

To further explore TME differences, we focused on the male subgroup. This analysis confirmed marked shifts in overall cell type composition, with HPV+ tumors showing increased abundance of B cells and mast cells, whereas HPV- tumors were enriched for monocytes, macrophages, and fibroblasts, patterns indicative of a more inflammatory and stromal-rich TME in HPV- tumors. To further dissect immune function, we examined pathway-level activity in CD4^+^ and CD8^+^ T cells using UCell scoring. Interestingly, both cytotoxic CD8^+^ T cell and Treg signatures were significantly elevated in HPV- tumors. This finding is unexpected, as previous studies have typically reported stronger CD8-mediated cytotoxic responses in HPV^+^ tumors, likely driven by viral antigenicity.^28^ The discrepancy may stem from cohort-specific differences, integration strategy, signature selection, or sample handling, and highlights the importance of stratified and context-aware analyses. Moreover, the concurrent elevation of cytotoxic and regulatory programs may reflect a context-dependent inflammatory state rather than a uniformly activated immune phenotype. These findings warrant validation in independent cohorts. Although the limited number of female patients did not allow for robust statistical comparisons, preliminary trends suggest that HPV- females may harbor a distinct TME, potentially shaped by sex-specific immune regulation. These observations, together with prior studies highlighting sex differences in TME composition and immune behavior, point to the need for future studies with balanced cohorts to explore whether female patients could benefit from tailored therapeutic approaches.^29^^,^^30^

Despite the high resolution of the single-cell atlas, scRNA-seq has inherent technical limitations, including dropouts and sampling bias, which may obscure certain biological signals. Additionally, the high cost and resource-intensive nature of single-cell technologies can limit accessibility, potentially introducing biases in sample selection.^31^ On the computational side, analyzing large single-cell datasets require substantial storage, computational power, and continuously evolving bioinformatics tools to manage the growing complexity of data. Furthermore, the study design itself can introduce biases, as seen in this atlas, where the proportion of male and female patients is unbalanced, potentially affecting the interpretation of sex-related differences. This limitation, together with the evolving landscape of available datasets, highlights the importance of ongoing updates. While this atlas integrates the most comprehensive single-cell data available at the time of analysis, recent studies, such as those by Xiong et al. and Kim et al., have since added valuable transcriptomic information.^32^^,^^33^ However, due to the absence of key clinical annotations such as HPV status and the lack of detectable HPV transcripts, they were not suitable for inclusion in our stratified framework. However, the open and modular pipeline allows for the future incorporation of such datasets in complementary analyses. These challenges and opportunities highlight the need for continued refinement of both experimental design and analytical strategies to fully leverage the potential of single-cell data in understanding tumor biology.

One promising avenue to overcome these limitations is the integration of multi-omics data, incorporating genomics, transcriptomics, proteomics, and metabolomics to provide a more comprehensive understanding of the TME. Combining scRNA-seq with spatial transcriptomics or epigenetic profiling, for instance, we could enhance the resolution of tumor-immune interactions and identify regulatory mechanisms shaping immune responses in HNSCC.^34^ Recent multi-omics studies have demonstrated the power of such approaches: For example, Ren et al. integrated genomic, transcriptomic, and proteomic data in hypopharyngeal squamous cell carcinoma and revealed sex-biased molecular programs and HPV-associated prognostic signatures that were not apparent from single-modality analyses alone. These findings highlight how multi-layered profiling can uncover biologically and clinically relevant sex differences in HNC subtypes.^35^ Additionally, longitudinal studies tracking tumor evolution over time would provide valuable insights into tumor heterogeneity, adaptation to treatment, and immune evasion. Addressing these challenges while maintaining a robust integrative approach, such as our unified annotation atlas, will be essential for improving the development of effective, personalized therapies in HNSCC.

In summary, this study highlights the value of large-scale data integration in advancing the understanding of the TME in HNSCC. By creating a unified single-cell atlas, we synthesized data from diverse patient transcriptomic profiles, overcoming the inconsistencies that often hinder cross-study comparisons. This integrative approach enhances statistical power by incorporating a larger number of samples, allowing for more refined stratification and robust subgroup analyses. With comprehensive clinical annotations such as sex, HPV status, and environmental exposures (e.g., alcohol or tobacco), the atlas allows a deeper exploration of factors shaping the TME and addresses a broader range of research questions.

Single-cell transcriptomics has opened up new opportunities to map the TME in significant detail. By integrating data from 78 patients, we created a unified HNSCC atlas that allowed us to explore key factors such as sex, HPV status, and immune and stromal composition on a much larger scale than was previously possible. This integration not only overcomes limitations of isolated studies but also sheds light on distinct immune and stromal profiles across these variables, offering insights into tumor biology that were previously unattainable for HNSCC.

Our atlas enables stratified exploration of HPV status and sex as key variables shaping the TME, highlighting trends that merit validation in future, balanced cohorts. In this context, sex emerges as an important and often underexplored biological variable that warrants further investigation in adequately powered studies. By providing a harmonized and publicly available resource, this work facilitates reproducible investigation of HNSCC biology and supports future studies aimed at refining patient stratification and therapeutic strategies.

This atlas is publicly available to ensure its utility as a resource for the research community, facilitating reproducibility and enabling the exploration of new hypotheses in HNSCC. Although developed in the context of HNSCC, the integrative framework combining STACAS-based harmonization with automated tumor and immune cell annotation is broadly applicable to other highly heterogeneous diseases, such as lung or esophageal cancer. Adaptation to additional tumor types would primarily require tumor-specific gene signatures and appropriate clinical annotation, rather than fundamental changes to the pipeline itself. Moving forward, this integrative approach underscores the importance of harmonized datasets for advancing our understanding of tumor heterogeneity and microenvironmental regulation.

### Limitations of the study

This study integrates the most comprehensive publicly available HNSCC single-cell RNA-seq datasets that met our inclusion criteria, but several limitations should be considered. First, the cohort is imbalanced by sex, with a strong predominance of male patients and only two HPV-positive female samples, which limits statistical power for sex-stratified analyses and means that these findings should be interpreted as exploratory. Second, the atlas combines datasets generated with different sample processing workflows, sequencing depths, and study designs, which may leave residual technical and biological heterogeneity despite harmonized integration. Third, single-cell RNA-seq has inherent technical limitations, including dropout events and sampling bias, that may obscure low-abundance populations or transient cellular states. Finally, some newer datasets could not be included because key clinical annotations, such as HPV status, were unavailable or HPV transcripts were not detectable, which restricts the scope of stratified analyses.

## Resource availability

### Lead contact

Further information and requests for resources should be directed to and will be fulfilled by the lead contact, Cristina Conde López (cristina.condelopez@dkfz-heidelberg.de).

### Materials availability

This study did not generate new biological materials.

### Data and code availability

• **Data:** The integrated HNSCC single-cell atlas generated in this study is publicly available through the HNSCatlas R package at https://github.com/DKFZ-E220/HNSCatlas. The curated atlas object (.rds format) is also deposited in the Helmholtz HIFIS repository at https://hifis-storage.desy.de:2880/Helmholtz/E220-Radioonc_biol-DKFZ/HNSCC_Atlas.rds. Any additional data reported in this paper will be shared by the lead contact upon request.
• **Code:** All scripts used for preprocessing, integration, annotation, inferCNV analysis, UCell scoring, and CoVarNet-based analyses are available in the GitHub repository (inst/extdata directory). The software resources used in the study are listed in the key resources table with source and version information. No new custom algorithms were developed for this study.
• **Other:** Any additional information required to reanalyze the data reported in this paper is available from the lead contact upon request.

## Acknowledgments

We appreciate the kind funding support from the DKFZ-MOST (Israeli Ministry of Science and Technology) Program in Cancer Research.

## Author contributions

C.C.L. conceptualized the study, developed the methodology, performed the formal analysis, conducted data curation, generated visualizations, and wrote the original draft of the manuscript. W.W.H., R.H., and D.M. contributed to methodology development and data analysis. M.J.B. and M.R. contributed to validation and critical revision. M.E., J.H., and I.K. contributed to conceptual guidance, supervision, and critical revision of the manuscript. Funding acquisition was provided by M.E, I.K., and J.H. All authors reviewed and approved the final manuscript.

## Declaration of interests

The authors declare no competing interests.

## Declaration of generative AI and AI-assisted technologies in the writing process

During the preparation of this work, the authors used ChatGPT (OpenAI) to enhance text readability, clarity, and flow. After using this tool, the authors reviewed and edited the content as needed and take full responsibility for the content of the published article.

## STAR★Methods

### Key resources table

**Table:** 

REAGENT or RESOURCE	SOURCE	IDENTIFIER
**Deposited data**		

HNSCC scRNA-seq dataset	GEO	GSE234933
HNSCC scRNA-seq dataset	GEO	GSE182227
HNSCC scRNA-seq dataset	GEO	GSE164690
HNSCC scRNA-seq dataset	GEO	GSE181919
ESCC scRNA-seq dataset	GEO	GSE160269
Integrated HNSCC atlas (R Object)	Helmholtz HIFIS Repository	https://hifis-storage.desy.de:2880/Helmholtz/E220-Radioonc_biol-DKFZ/HNSCC_Atlas.rds
HNSCAtlas R package repository	GitHub	https://github.com/DKFZ-E220/HNSCatlas

**Software and algorithms**		

R	The R Foundation	v4.4.1
Seurat	Satija Lab/CRAN	v4.3
STACAS	Andreatta et al.^36^	https://github.com/carmonalab/STACAS
scGate	Andreatta et al.^37^	https://github.com/carmonalab/scGate
ProjectTILs signatures	Andreatta et al.^23^	https://github.com/carmonalab/ProjectTILS
Ikarus	Dohmen et al.^17^	https://github.com/BIMSBbioinfo/ikarus
Zellkonverter	Bioconductor	https://github.com/theislab/zellkonverter
inferCNV	Broad Institute	https://github.com/broadinstitute/infercnv
CoVarNet	Shi et al.^38^	https://github.com/QiangShiPKU/CoVarNet
UCell	Andreatta & Carmona^39^	https://github.com/carmonalab/UCell
Python	Python Software Foundation	v3.8

**Other**		

Human reference genome (GRCh38/hg38)	Genome Reference Consortium	GRCh38

### Experimental model and study participant details

A comprehensive search was conducted for publicly available HNSCC scRNA-seq datasets. The datasets, GEO: GSE234933, GEO: GSE182227, GEO: GSE164690, and GEO: GSE181919, respectively, were selected from the Gene Expression Omnibus (GEO) platform based on having appropriate clinical annotations; these include sex, HPV status, and the origin of the sample (normal tissue, primary tumor, or metastasis).^2^^,^^16^^,^^28^^,^^40^ Notably, GEO: GSE164690 included both viable CD45^+^ immune cells and CD45^−^non-immune tumor/stromal cells following fluorescence-based sorting; both compartments were incorporated into the integration framework. Prior to integration, a critical pruning process was applied to these datasets, which involved removing data points with missing essential annotations (sex, HPV status, or sample origin) and renaming variables to standardize terminology across studies. This ensured consistency and alignment in data structure, enabling more robust comparisons, and a solid foundation for accurate integration of the datasets. The final integrated dataset consists of 78 patients with complete sex and HPV status annotations, including 46 HPV- cases (32 male, 14 female) and 32 HPV+ cases (30 male, 2 female). In total, 274,911 cells were retained after quality control filtering and exclusion of samples lacking complete clinical metadata. Cell numbers per patient varied across cohorts (range: 282–10,667 cells; median: 3,246), reflecting differences in original sequencing depth and study design. All measurements derive from distinct biological samples across patients; no repeated measures were taken.

This study reanalyzed publicly available de-identified scRNA-seq datasets with appropriate ethical approvals from the original studies (GSE234933, GSE182227, GSE164690, and GSE181919). No new human subjects were recruited, and no identifiable patient information was used.

### Method details

#### Preprocessing and Seurat workflow

A standard workflow using the R package Seurat was performed on the combined HNSCC datasets.^41^ The Seurat functions help to adjust for differences in sequencing depth and technological biases between datasets. Normalization ensures that the data across different studies are compatible and can be integrated meaningfully, providing a reliable basis for comparison and further analysis. First, data normalization was performed using Seurat’s function NormalizeData(), which adjusts the expression measurements for each cell to allow for more accurate comparisons across different cells. Following normalization, the FindVariableFeatures() function was applied in order to identify the most variable genes across the dataset, which are crucial to distinguishing between cell types. Data were scaled with ScaleData() to ensure that highly variable genes do not overshadow the influence of genes with smaller variability.

#### Semi-supervised integration with Stacas

The R package Stacas, a semi-supervised method, was used to integrate single-cell datasets from multiple HNSCC studies.^36^ This approach employs semi-supervised labeling to align datasets while preserving biologically relevant differences, such as unique cell type distributions or expression patterns specific to each dataset. Run.STACAS() was applied to the preprocessed datasets to identify shared anchors, representing mutual biological similarities across cells. These anchors were then used to project cells from different datasets into a shared space, minimizing batch effects and maintaining key biological features. By leveraging both labeled and unlabeled data through the cell.labels parameter, Stacas improves accuracy by matching similar cell types across datasets. This method standardizes data from diverse sources while preserving biological integrity, enabling a cohesive and reliable analysis.

#### Cell type annotation with scGate

The R package scGate, an annotation tool, was employed, which automates the identification of cell types in the datasets using predefined gating models (GMs).^23^ These GMs are based on commonly used markers for immune cells in humans and mice, such as T cells, B cells, NK cells, and myeloid populations. The models are refined with data from five annotated datasets from studies on blood or tumor samples, which are already integrated into the package.^42^^,^^43^^,^^44^^,^^45^^,^^46^ This step ensured consistent and precise cell type classification across different datasets, essential for analyzing complex interactions within the TME.

#### Tumor cell annotation with Ikarus

The Ikarus method, a fully supervised classifier designed to detect tumor cells with high specificity was used to accurately annotate tumor cells.^17^ Ikarus applies pre-trained models on labeled gene signatures characteristic of tumor profiles, enabling precise tumor cell identification in complex cellular environments. The integrated Seurat object was prepared for Ikarus compatibility by converting it to the H5AD format with the DietSeurat() and zellkonverter::SCE2AnnData functions. By using Ikarus in Python, tumor cells were identified across the selected HNSCC datasets and the Ikarus-generated labels were integrated with the existing tumor cell annotations to enhance specificity and ensure reliable tumor cell identification.

#### Validation of annotation pipeline on an independent ESCC dataset

To assess the generalisability of the annotation pipeline, an independent esophageal squamous cell carcinoma (ESCC) dataset (GEO: GSE160269) was processed using the same framework described above. The dataset comprised negatively and positively sorted cell fractions, which were merged into a single Seurat object following standard quality control and normalisation. Cell type annotation was performed sequentially using scGate and Ikarus, following identical procedures to those applied in the HNSCC atlas. No cross-dataset integration was performed, as all samples originated from a single study. Annotation concordance with the original labels was assessed using a row-normalised overlap heatmap.

#### inferCNV analysis

To compare chromosomal copy number variation (CNV) profiles between epithelial cells, originally annotated tumor cells, and cells reclassified from epithelial to tumor, inferCNV R package was used.^47^ A subset of 4,818 cells (the limiting size of the relabeled epithelial group) was selected from each category for balanced analysis. Random sampling ensured equal representation across groups. The RNA count matrix was converted to a standard format, and cells were annotated into three categories: epithelial, tumor, and epithelial_tumor. The “Epithelial” group was used as the reference for CNV detection. Gene order was defined using hg38 coordinates, and a cutoff of 0.1 was applied to filter low-expression genes. Denoising and a Hidden Markov Model were used to infer CNV states. Analyses were performed on a high-memory computational cluster.

#### Immune subclassification

A detailed subclassification of immune cells was performed utilizing the ProjectTILS framework, which is encompassed within the R package scGate. It employs preestablished reference labeling for critical immune cell types such as dendritic cells, CD4, and CD8 T cells.

#### Co-variation network analysis using CoVarNet

The CoVarNet framework, a computational approach designed to identify higher-order coordination among cell types, was applied to characterize TME organization across HNSCC samples. CoVarNet quantifies the covariance in cell type proportions across patients to infer cellular co-variation modules (CMs), reflecting coordinated shifts in cell population abundances. For this analysis, the integrated single-cell atlas was used to compute per-patient cell type proportions, which were then provided as input to CoVarNet in R. The framework constructs a covariance matrix based on these proportions and performs hierarchical clustering to define modules of co-varying cell types. Each module was subsequently weighted by its relative contribution of cell types, and module abundance scores were computed for each patient.

### Quantification and statistical analysis

All analyses were performed on the integrated HNSCC single-cell atlas using patient-level comparisons where applicable. Per-patient cell type proportions were calculated from annotated cells, and GEO: GSE164690 was excluded from composition analyses because prior CD45 sorting could bias proportions, although it was retained for within-cell-type analyses such as UCell scoring. Group comparisons of proportions and signature scores were performed using Wilcoxon rank-sum tests, whereas projected count comparisons used Fisher’s exact test. Multiple-testing correction was performed using the Benjamini-Hochberg method where applicable, and *p* < 0.05 was considered significant unless otherwise stated. Integration quality was assessed using LISI and ASW, and agreement between original and relabeled annotations was evaluated using ARI and NMI.

### Additional resources

No additional resources are associated with this study.

## References

[1] Bray F., Ferlay J., Soerjomataram I., Siegel R.L., Torre L.A., Jemal A. (2018). Global cancer statistics 2018: GLOBOCAN estimates of incidence and mortality worldwide for 36 cancers in 185 countries. CA Cancer J. Clin..

[2] Puram S.V., Mints M., Pal A., Qi Z., Reeb A., Gelev K., Barrett T.F., Gerndt S., Liu P., Parikh A.S. (2023). Cellular states are coupled to genomic and viral heterogeneity in HPV-related oropharyngeal carcinoma. Nat. Genet..

[3] Mito I., Takahashi H., Kawabata-Iwakawa R., Ida S., Tada H., Chikamatsu K. (2021). Comprehensive analysis of immune cell enrichment in the tumor microenvironment of head and neck squamous cell carcinoma. Sci. Rep..

[4] Hollows R., Wei W., Cazier J.-B., Mehanna H., Parry G., Halford G., Murray P. (2019). Association between loss of Y chromosome and poor prognosis in male head and neck squamous cell carcinoma. Head Neck.

[5] Hunt K.S., Alspach E. (2024). Battle Within the Sexes: Differences in Male and Female Immunity and the Impact on Antitumor Responses. Cancer Immunol. Res..

[6] Jawa Y., Yadav P., Gupta S., Mathan S.V., Pandey J., Saxena A.K., Kateriya S., Tiku A.B., Mondal N., Bhattacharya J. (2021). Current Insights and Advancements in Head and Neck Cancer: Emerging Biomarkers and Therapeutics with Cues from Single Cell and 3D Model Omics Profiling. Front. Oncol..

[7] Cai Z., Tang B., Chen L., Lei W. (2022). Mast cell marker gene signature in head and neck squamous cell carcinoma. BMC Cancer.

[8] Edwards N.J., Oberti M., Thangudu R.R., Cai S., McGarvey P.B., Jacob S., Madhavan S., Ketchum K.A. (2015). The CPTAC Data Portal: A Resource for Cancer Proteomics Research. J. Proteome Res..

[9] Weinstein J.N., Collisson E.A., Mills G.B., Shaw K.R.M., Ozenberger B.A., Ellrott K., Shmulevich I., Sander C., Stuart J.M., Cancer Genome Atlas Research Network (2013). The Cancer Genome Atlas Pan-Cancer analysis project. Nat. Genet..

[10] Diao P., Dai Y., Wang A., Bu X., Wang Z., Li J., Wu Y., Jiang H., Wang Y., Cheng J. (2024). Integrative multiomics analyses identify molecular subtypes of head and neck squamous cell carcinoma with distinct therapeutic vulnerabilities. Cancer Res..

[11] Stampe H., Jakobsen K.K., Bendtsen S.K., Grønhøj C., von Buchwald C. (2021). Systematic review on the current knowledge and use of single-cell RNA sequencing in head and neck cancer. APMIS.

[12] Huang Z.-D., Liu Z.-Z., Liu Y.-Y., Fu Y.-C., Lin L.-L., Hu C., Gu H.-Y., Wei R.-X. (2021). Molecular subtypes based on cell differentiation trajectories in head and neck squamous cell carcinoma: Differential prognosis and immunotherapeutic responses. Front. Immunol..

[13] Perou C.M., Sørlie T., Eisen M.B., van de Rijn M., Jeffrey S.S., Rees C.A., Pollack J.R., Ross D.T., Johnsen H., Akslen L.A. (2000). Molecular portraits of human breast tumours. Nature.

[14] Conde-Lopez C., Marripati D., Elkabets M., Hess J., Kurth I. (2024). Unravelling the complexity of HNSCC using single-cell transcriptomics. Cancers (Basel).

[15] Qi Z., Barrett T., Parikh A.S., Tirosh I., Puram S.V. (2019). Single-cell sequencing and its applications in head and neck cancer. Oral Oncol..

[16] Bill R., Wirapati P., Messemaker M., Roh W., Zitti B., Duval F., Kiss M., Park J.C., Saal T.M., Hoelzl J. (2023). CXCL9:SPP1 macrophage polarity identifies a network of cellular programs that control human cancers. Science.

[17] Dohmen J., Baranovskii A., Ronen J., Uyar B., Franke V., Akalin A. (2022). Identifying tumor cells at the single-cell level using machine learning. Genome Biol..

[18] Yu X., Wang Z., Zeng T. (2020). Essential gene expression pattern of head and neck squamous cell carcinoma revealed by tumor-specific expression rule based on single-cell RNA sequencing. Biochim. Biophys. Acta Mol. Basis Dis..

[19] Yu X., Chen Y.A., Conejo-Garcia J.R., Chung C.H., Wang X. (2019). Estimation of immune cell content in tumor using single-cell RNA-seq reference data. BMC Cancer.

[20] Zappia L., Theis F.J. (2021). Over 1000 tools reveal trends in the single-cell RNA-seq analysis landscape. Genome Biol..

[21] Heumos L., Schaar A.C., Lance C., Litinetskaya A., Drost F., Zappia L., Lücken M.D., Strobl D.C., Henao J., Curion F. (2023). Best practices for single-cell analysis across modalities. Nat. Rev. Genet..

[22] Cords L., Tietscher S., Anzeneder T., Langwieder C., Rees M., de Souza N., Bodenmiller B. (2023). Cancer-associated fibroblast classification in single-cell and spatial proteomics data. Nat. Commun..

[23] Andreatta M., Corria-Osorio J., Müller S., Cubas R., Coukos G., Carmona S.J. (2021). Interpretation of T cell states from single-cell transcriptomics data using reference atlases. Nat. Commun..

[24] Zhang X., Peng L., Luo Y., Zhang S., Pu Y., Chen Y., Guo W., Yao J., Shao M., Fan W. (2021). Dissecting esophageal squamous-cell carcinoma ecosystem by single-cell transcriptomic analysis. Nat. Commun..

[25] Liu S., Wang R., Fang J. (2024). Exploring the frontiers: tumor immune microenvironment and immunotherapy in head and neck squamous cell carcinoma. Discov. Oncol..

[26] Dai Y., Wang Z., Xia Y., Li J., Wu Y., Wang Y., Jiang H., Cheng J. (2023). Integrative single-cell and bulk transcriptomes analyses identify intrinsic HNSCC subtypes with distinct prognoses and therapeutic vulnerabilities. Clin. Cancer Res..

[27] Ruffin A.T., Li H., Vujanovic L., Zandberg D.P., Ferris R.L., Bruno T.C. (2023). Improving head and neck cancer therapies by immunomodulation of the tumour microenvironment. Nat. Rev. Cancer.

[28] Kürten C.H.L., Kulkarni A., Cillo A.R., Santos P.M., Roble A.K., Onkar S., Reeder C., Lang S., Chen X., Duvvuri U. (2021). Investigating immune and non-immune cell interactions in head and neck tumors by single-cell RNA sequencing. Nat. Commun..

[29] Ning K., Peng Y., Jiang Y., Li Z., Luo X., Lin L., Deng M., Wu Y., Huang T., Huang Y. (2023). Sex differences in renal cell carcinoma: a single-cell analysis reveals exhausted CD8+ T-cells highly infiltrated in males. Biol. Sex Differ..

[30] Yan H., Huang J., Li Y., Zhao B. (2024). Sex disparities revealed by single-cell and bulk sequencing and their impacts on the efficacy of immunotherapy in esophageal cancer. Biol. Sex Differ..

[31] Gondal M.N., Shah S.U.R., Chinnaiyan A.M., Cieslik M. (2024). A systematic overview of single-cell transcriptomics databases, their use cases, and limitations. Front. Bioinform..

[32] Xiong M., Hu J.-J., Yao M.-L., Song T.-T., Zhao L., Mou B.-Q., Qian Y.-X., Zheng M.-J., Dong Y.-J., Wang H.-Y. (2024). Single-cell sequencing of head and neck carcinoma: Transcriptional landscape and prognostic model based on malignant epithelial cell features. FASEB J..

[33] Kim S., Kee H.J., Kim D., Jang J., Jeong H.-O., Sim N.S., Selig M., Ihlow J., Penter L., Hwang T. (2025). Multiregional single-cell transcriptomics reveals an association between partial EMT and immunosuppressive states in oral squamous cell carcinoma. iScience.

[34] Li X., Wang C.-Y. (2021). From bulk, single-cell to spatial RNA sequencing. Int. J. Oral Sci..

[35] Ren Y., Xiong W., Feng C., Yu D., Wang X., Yang Q., Yu S., Zhang H., Huo B., Jiang H. (2025). Multi-omics insights into the molecular signature and prognosis of hypopharyngeal squamous cell carcinoma. Commun. Biol..

[36] Andreatta M., Hérault L., Gueguen P., Gfeller D., Berenstein A.J., Carmona S.J. (2024). Semi-supervised integration of single-cell transcriptomics data. Nat. Commun..

[37] Andreatta M., Berenstein A.J., Carmona S.J. (2022). scGate: marker-based purification of cell types from heterogeneous single-cell RNA-seq datasets. Bioinformatics.

[38] Shi Q., Chen Y., Li Y., Qin S., Yang Y., Gao Y., Zhu L., Wang D., Zhang Z. (2025). Cross-tissue multicellular coordination and its rewiring in cancer. Nature.

[39] Andreatta M., Carmona S.J. (2021). UCell: Robust and scalable single-cell gene signature scoring. Comput. Struct. Biotechnol. J..

[40] Puram S.V., Tirosh I., Parikh A.S., Patel A.P., Yizhak K., Gillespie S., Rodman C., Luo C.L., Mroz E.A., Emerick K.S. (2017). Single-cell transcriptomic analysis of primary and metastatic tumor ecosystems in head and neck cancer. Cell.

[41] Hao Y., Stuart T., Kowalski M.H., Choudhary S., Hoffman P., Hartman A., Srivastava A., Molla G., Madad S., Fernandez-Granda C., Satija R. (2024). Dictionary learning for integrative, multimodal and scalable single-cell analysis. Nat. Biotechnol..

[42] Abdelaal T., Michielsen L., Cats D., Hoogduin D., Mei H., Reinders M.J.T., Mahfouz A. (2019). A comparison of automatic cell identification methods for single-cell RNA sequencing data. Genome Biol..

[43] Hao Y., Hao S., Andersen-Nissen E., Mauck W.M., Zheng S., Butler A., Lee M.J., Wilk A.J., Darby C., Zager M. (2021). Integrated analysis of multimodal single-cell data. Cell.

[44] Jerby-Arnon L., Shah P., Cuoco M.S., Rodman C., Su M.-J., Melms J.C., Leeson R., Kanodia A., Mei S., Lin J.-R. (2018). A cancer cell program promotes T cell exclusion and resistance to checkpoint blockade. Cell.

[45] Yost K.E., Satpathy A.T., Wells D.K., Qi Y., Wang C., Kageyama R., McNamara K.L., Granja J.M., Sarin K.Y., Brown R.A. (2019). Clonal replacement of tumor-specific T cells following PD-1 blockade. Nat. Med..

[46] Zilionis R., Engblom C., Pfirschke C., Savova V., Zemmour D., Saatcioglu H.D., Krishnan I., Maroni G., Meyerovitz C.V., Kerwin C.M. (2019). Single-cell transcriptomics of human and mouse lung cancers reveals conserved myeloid populations across individuals and species. Immunity.

[47] Tickle T., Tirosh I., Georgescu C., Brown M., Haas B. (2019). https://github.com/broadinstitute/inferCNV.

