# Supplementary material for: A unified single-cell atlas of HNSCC: Toward characterizing HPV- and sex-associated TME variability
Source: iScience. 2026 Apr 22;29(6):115863. doi: 10.1016/j.isci.2026.115863 (PMC13191048; doi:10.1016/j.isci.2026.115863)
Supplement: Document S1. Figures S1–S5 and Tables S1–S8 [file mmc1.pdf]

iScience, Volume *29*

Supplemental information

A unified single-cell atlas of HNSCC: Toward characterizing HPV- and sex-associated TME variability

Cristina Conde-Lopez, Divyasree Marripati, Maria Jose Besso, Mareike Roscher, Rui Han, Wahyu Wijaya Hadiwikarta, Moshe Elkabets, Jochen Hess, and Ina Kurth

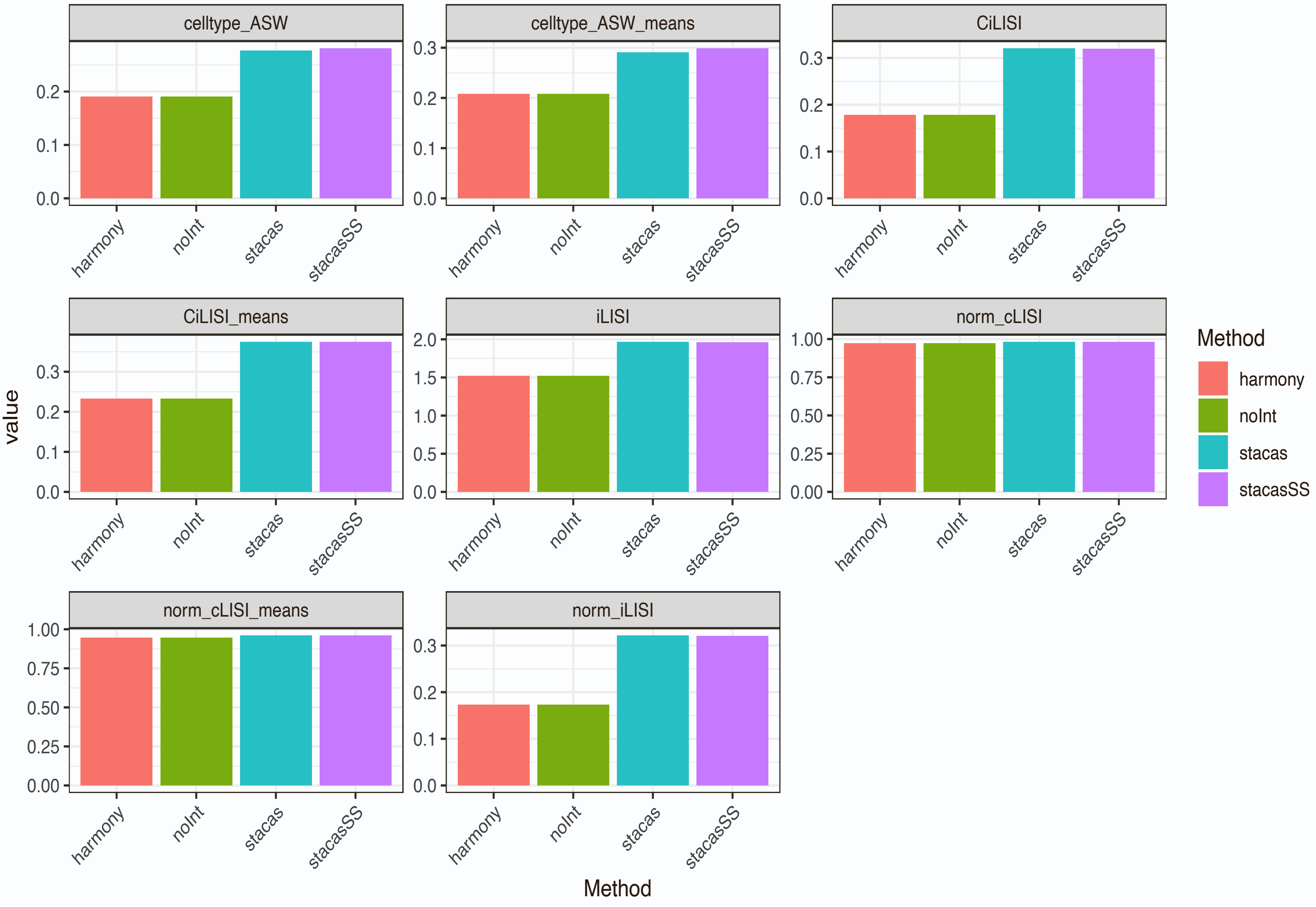

Supplementary Figure 1. Evaluation of integration quality across methods using batch mixing and cell identity preservation metrics.

This figure compares the performance of different data integration strategies, Harmony, no integration (noInt), STACAS, and STACAS with semi-supervised anchoring (STACAS-SS), based on a range of established metrics. These include cell type Adjusted Silhouette Width (ASW), cell-type and integration-specific Local Inverse Simpson's Index (cLISI and iLISI), and their normalized and averaged values. STACAS and STACAS-SS consistently achieve higher iLISI and ASW scores.

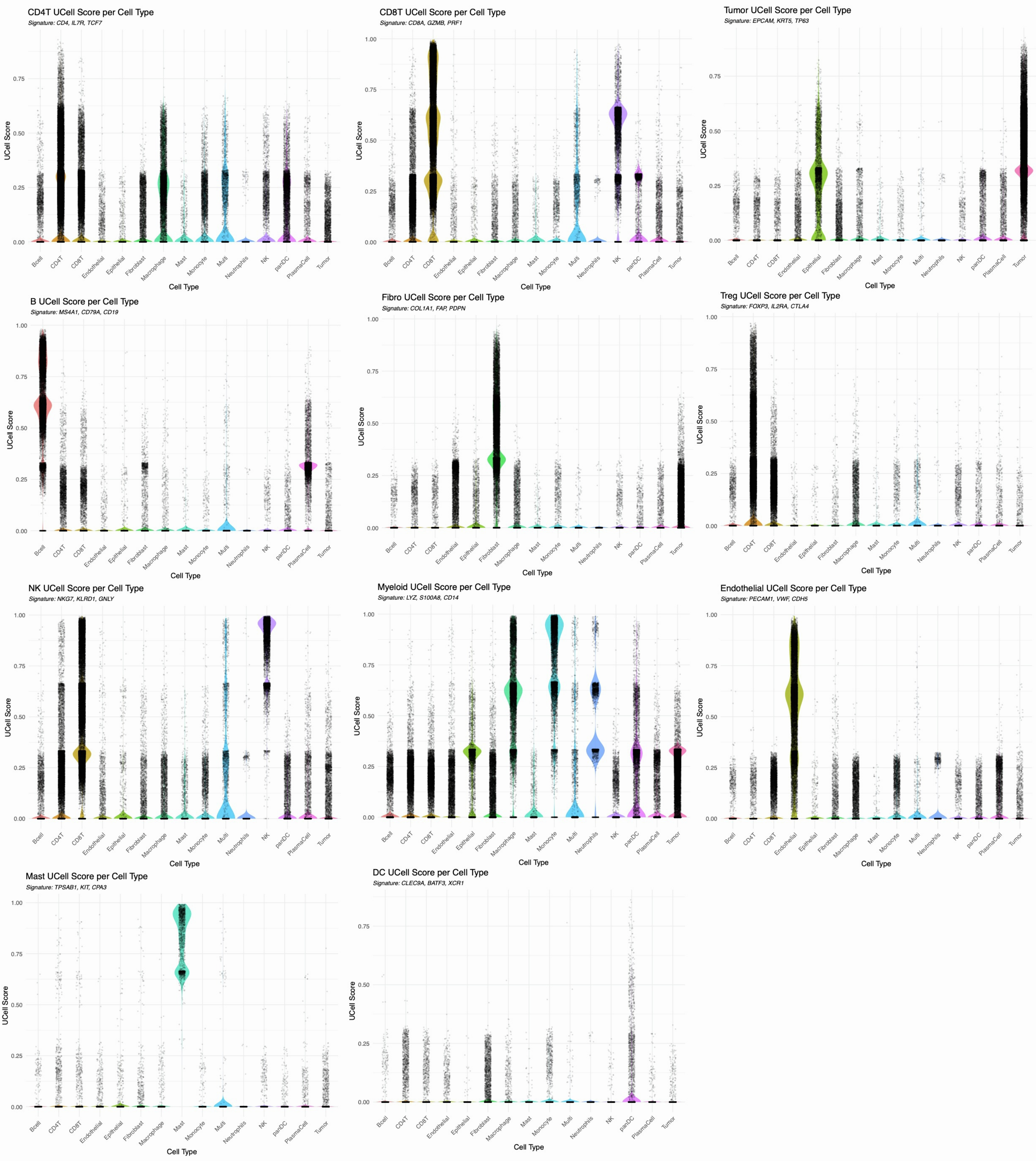

Supplementary Figure 2. Cell type specific gene signature scoring across annotated cell populations. Violin plots showing the expression of curated gene signatures across the final annotated cell types in the HNSCC atlas. Each panel corresponds to a distinct cell type signature (e.g., $CD8^+$ T cells, $CD4^+$ T cells, B cells, tumor cells or fibroblasts), and demonstrates strong enrichment of the corresponding score within the expected population.

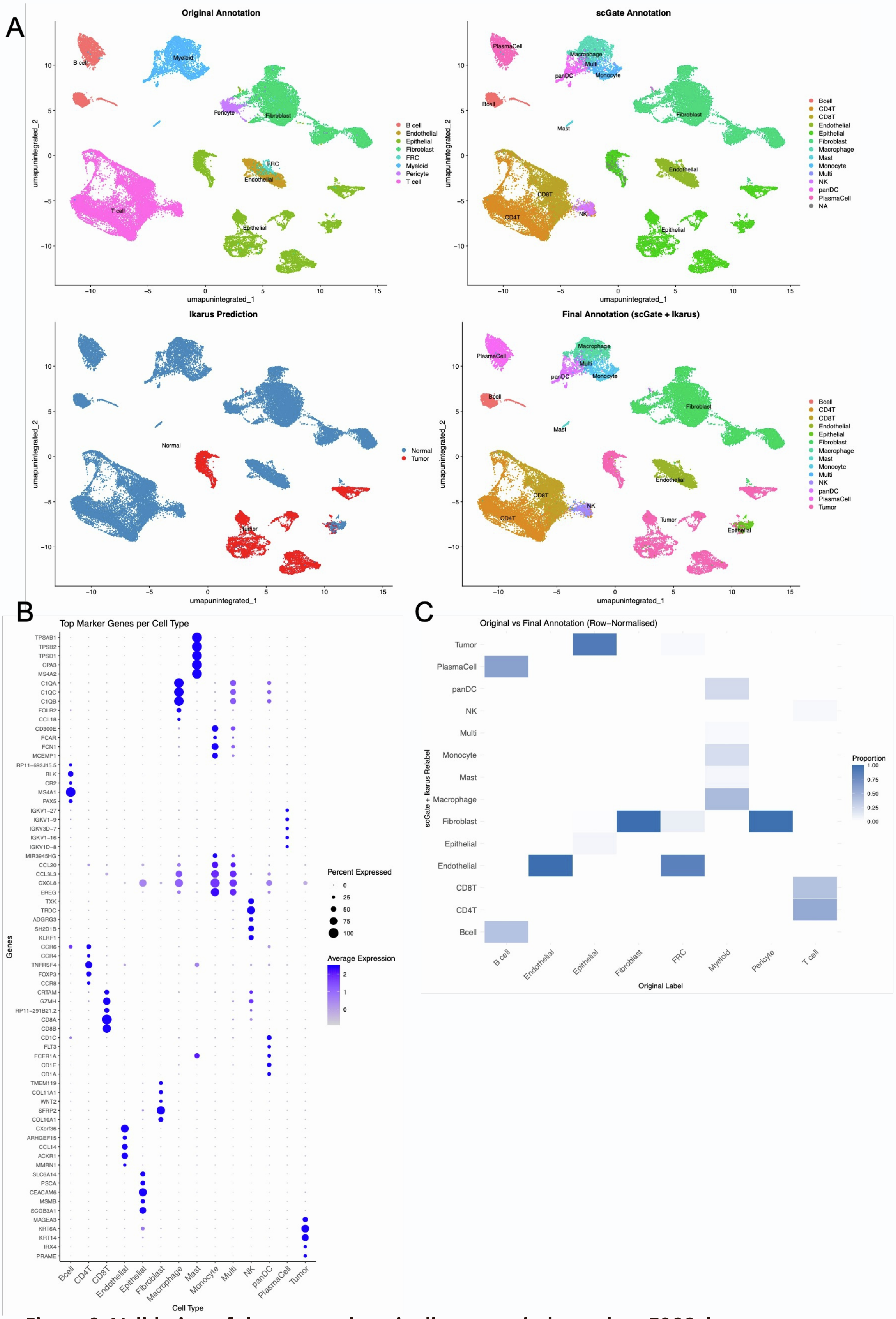

Supplementary Figure 3. Validation of the annotation pipeline on an independent ESCC dataset.

(A) UMAP plots showing cell type annotations before (Original Annotation) and after (scGate Annotation) applying the scGate TME_HiRes model, alongside Ikarus tumor cell predictions and the final combined annotation (scGate + Ikarus). The original annotation captures broad cell populations including B cells, T cells, epithelial, fibroblast, and myeloid cells. scGate refines these into consistent TME categories, while Ikarus identifies tumor cells independently based on gene expression signatures. The final annotation integrates both, replacing epithelial calls with tumor where supported by Ikarus. **(B)** Dot plot showing top differentially expressed marker genes per annotated cell type in the final annotation. Dot size reflects the percentage of cells expressing each gene and dot colour indicates average expression level. Key markers include PRAME and KRT14 for tumor cells, CD8A and GZMH for CD8 T cells, FOXP3 and CCR8 for CD4 T cells, and C1QA/C1QB for macrophages, confirming biological specificity of the annotations. **(C)** Row-normalised overlap heatmap comparing original and final (scGate + Ikarus) cell type labels. Each row represents an original label and colour intensity reflects the proportion of cells reassigned to each final category, highlighting consistent mapping between annotation frameworks.

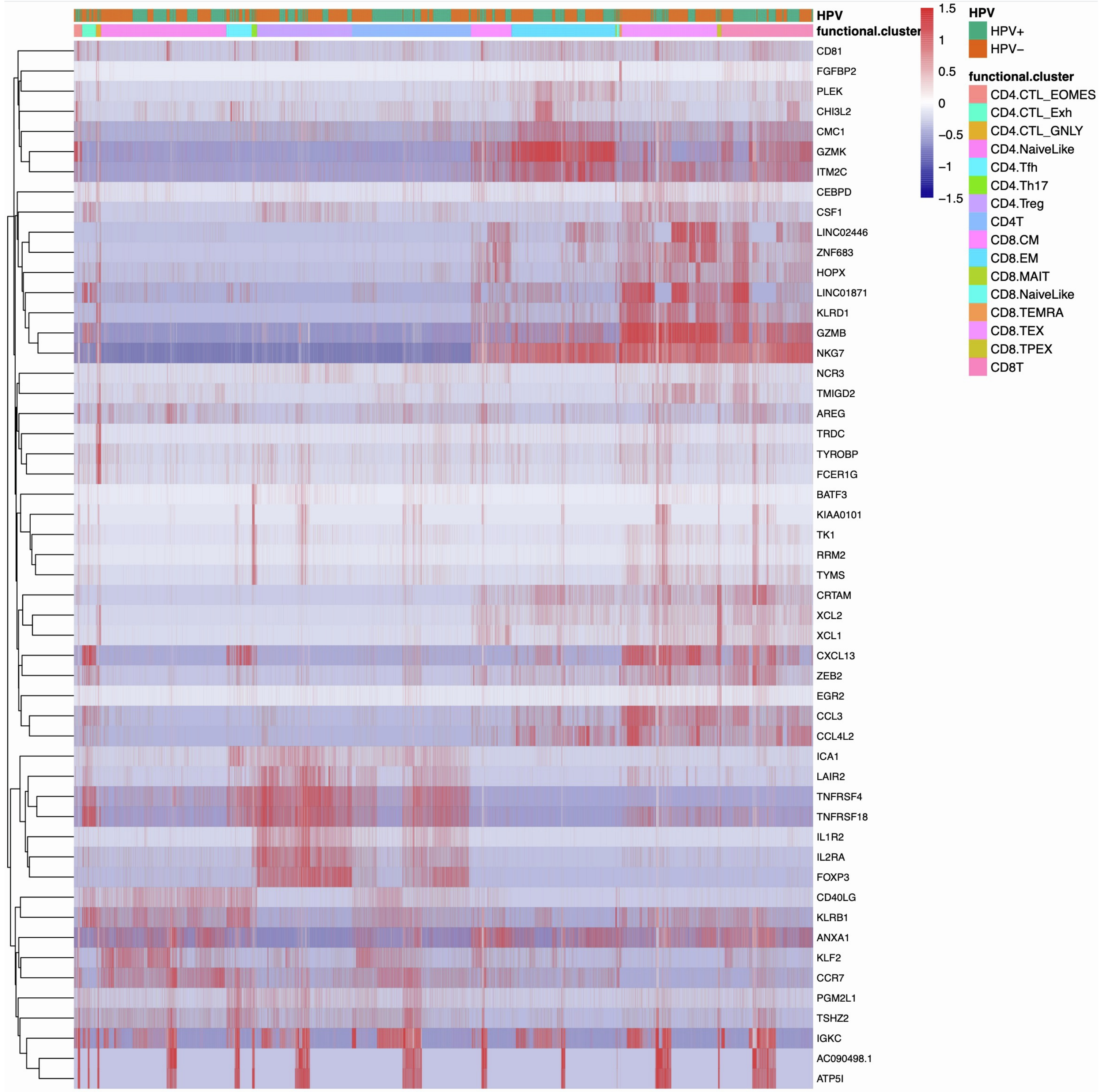

Supplementary Figure 4. Expression of top markers across annotated T cell subtypes.
This heatmap displays the scaled expression of the top differentially expressed genes across the annotated T cell clusters. Marker genes associated with cytotoxicity (e.g., GZMB, GZMK, NKG7), exhaustion (e.g., CXCL13, KLRD1), regulatory T cells (e.g., TNFRSF4, IL2RA, FOXP3), and helper T cell subsets (e.g., CXCL13, CD40LG, CCR7) show selective enrichment in their corresponding cell populations.

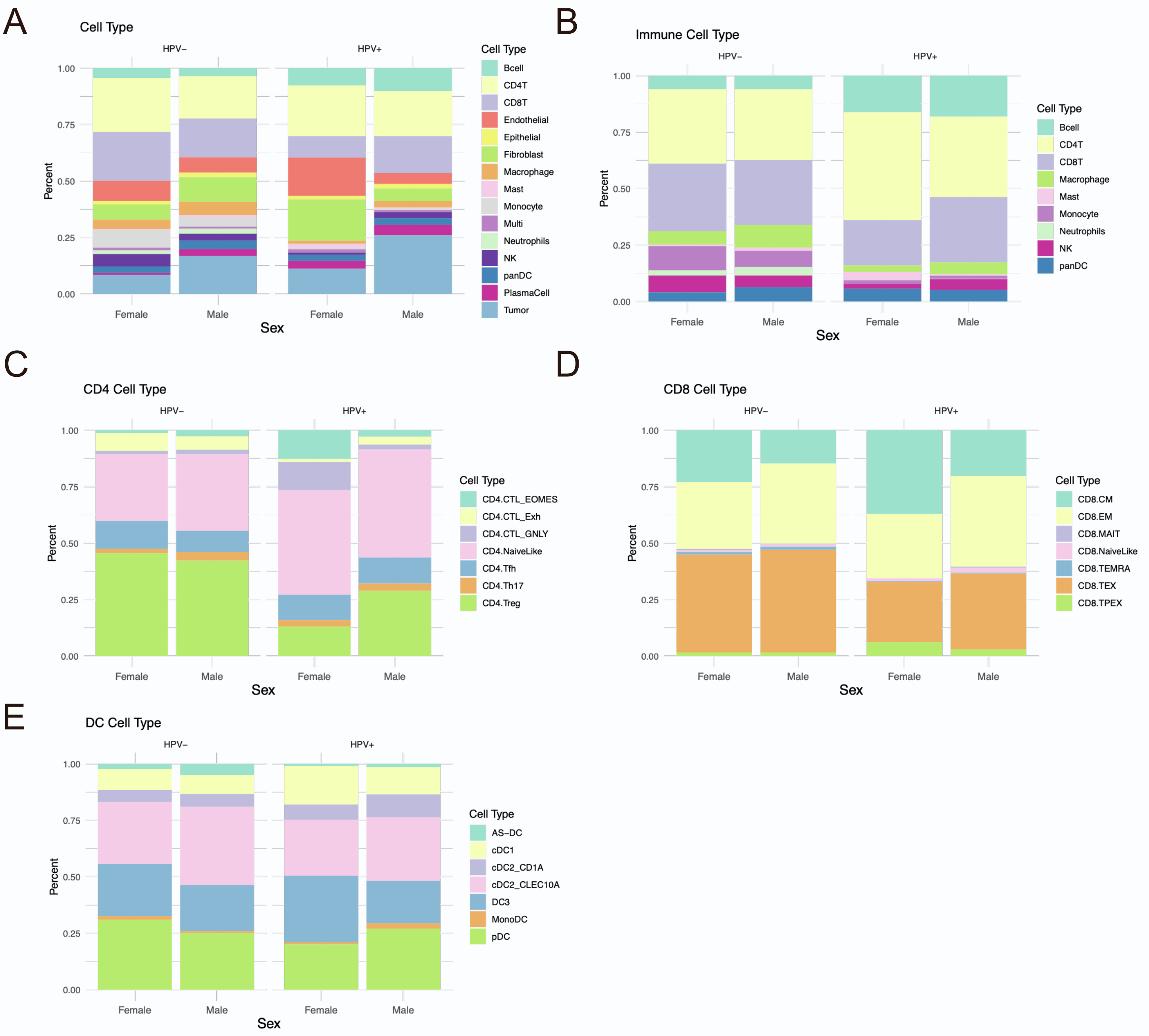

Supplementary Figure 5. Cell type composition in HNSCC tumors by sex, stratified by HPV status.
(A) Stacked bar plot showing the proportion of each major cell type across female and male groups, separated by HPV status (HPV+ and HPV-). The plot highlights sex differences in immune and stromal cell distributions. **(B)** Stacked bar plot focusing on overall immune cell types, showing distribution differences between females and males in HPV+ and HPV- groups. **(C)** Distribution of CD4+ T cell subtypes across sex and HPV status. **(D)** Distribution of CD8+ T cell subtypes across sex and HPV status.**(E)** Distribution of dendritic cell (DC) subtype composition across sex and HPV status.

Supplementary Table 1. Overview of publicly available HNSCC single-cell RNA sequencing datasets screened for atlas integration. The table summarizes accession number, year of publication, tissue type, number of samples, and total number of single cells reported for each dataset. Datasets were evaluated for inclusion based on availability of essential clinical metadata (sex and HPV status), sample composition, and suitability for cross-cohort integration. The four datasets included in the unified atlas are indicated, while excluded datasets are accompanied by the primary reason for exclusion.

Accession Number	*Year*	*Publication*	*Tissue Type*	*Sample Number*	*Approx. Cell Number*	*Included in Atlas*	*Reason for Exclusion (if excluded)*
**GSE234933**	2023	Bill et al., Science	Primary tumor, recurrence, metastasis	52	87399	Yes	—
**GSE182227**	2022	Puram et al., Nat Genet	Primary tumor, normal tissue	24	70970	Yes	—
**GSE164690**	2021	Kürten et al., Nat Commun	Primary tumor, PBMC	51	134606	Yes	—
**GSE181919**	2022	Choi et al., Nat Commun	Tumor, normal tissue, leukoplakia, LN metastasis	37	54239	Yes	—
**GSE139324**	2019	Cillo et al., Immunity	CD45+ immune populations	63	131224	No	Immune-only composition
**GSE200996**	2022	Luoma et al., Cell	Peripheral & intratumoral CD45+	204	74557	No	Immune-only composition
**GSE153559**	2020	Wieland et al., Nature	B cells, tumor, LN, periphery	7	8271	No	Immune-only composition
**GSE180268**	2021	Eberhardt et al., Nature	TILs	39	—	No	Immune-only composition
**GSE162025**	2020	Liu et al., Nat Commun	Primary tumor	40	176447	No	HPV-status unknown
**GSE150321**	2020	Song et al., Int J Cancer	Primary tumor	2	12985	No	HPV-status unknown
**GSE173647**	2022	—	Primary tumor	2	13903	No	HPV-status unknown
**GSE195832**	2022	Obradovic et al., Clin Cancer Res	Primary tumor	8	22906	No	Stromal-only composition
**GSE140042**	2021	—	Tumor, LN metastasis	9	—	No	HPV/sex -status unknow
**GSE213047**	2022	Lin et al., NPJ Precis Oncol	Tumor, normal, LN	3	11470	No	HPV/sex -status unknow
**GSE172577**	2021	Peng et al., Oral Oncol	Primary tumor	6	—	No	Immune-only composition

Supplementary Table 2. Clinical and technical characteristics of HNSCC patient samples included in the single-cell atlas. The table includes information on patient sex, HPV status, dataset of origin, total number of single cells retained after filtering, and tissue source (e.g., primary tumor, lymph node, or other anatomical location).

Patient	Sex	HPV	Cohort	Total_cells	Origin
BHN1	Male	HPV-	GSE234933	2568	Primary
BHN17	Male	HPV-	GSE234933	5756	Primary
BHN30	Male	HPV-	GSE234933	4420	Primary
BHN31TS	Male	HPV-	GSE234933	3868	Primary
BHN39	Male	HPV-	GSE234933	1272	Primary
BHN40-3-05	Female	HPV-	GSE234933	1735	Primary
BHN46	Female	HPV-	GSE234933	4181	Primary
BHN49	Male	HPV-	GSE234933	3254	Primary
BHN50	Male	HPV+	GSE234933	346	Primary
BHN52	Male	HPV+	GSE234933	1921	Primary
BHN58	Male	HPV+	GSE234933	2818	Primary
BHN59	Male	HPV+	GSE234933	5046	Primary
BHN60	Female	HPV-	GSE234933	4932	Primary
BHN63	Male	HPV+	GSE234933	8330	Primary
BHN64	Male	HPV-	GSE234933	4774	Primary
BHN67	Male	HPV-	GSE234933	3643	Primary
BHN68	Male	HPV+	GSE234933	8450	Primary
BHN7	Male	HPV-	GSE234933	1081	Primary
BHN70	Male	HPV-	GSE234933	4739	Primary
BHN72	Male	HPV-	GSE234933	3962	Primary
BHN74	Female	HPV-	GSE234933	9194	Primary
BHN75	Male	HPV-	GSE234933	6981	Primary
BHN76	Male	HPV+	GSE234933	6685	Primary
BHN77	Male	HPV+	GSE234933	7817	Primary
HN01	Male	HPV-	GSE164690	3473	Primary
HN02	Female	HPV-	GSE164690	748	Primary
HN03	Male	HPV-	GSE164690	5115	Primary
HN04	Male	HPV-	GSE164690	1383	Primary
HN05	Female	HPV-	GSE164690	5111	Primary
HN06	Male	HPV-	GSE164690	2667	Primary
HN07	Male	HPV-	GSE164690	3139	Primary
HN08	Female	HPV-	GSE164690	2289	Primary
HN09	Female	HPV-	GSE164690	3701	Primary
HN10	Male	HPV-	GSE164690	4465	Primary
HN11	Male	HPV-	GSE164690	4762	Primary
HN12	Male	HPV+	GSE164690	4543	Primary
HN13	Male	HPV+	GSE164690	5284	Primary
HN14	Male	HPV+	GSE164690	3720	Primary
HN15	Female	HPV-	GSE164690	4472	Primary
HN16	Male	HPV+	GSE164690	5086	Primary
HN17	Male	HPV+	GSE164690	10667	Primary
HN18	Male	HPV+	GSE164690	2455	Primary
OP10	Male	HPV-	GSE182227	7078	Primary
OP12	Male	HPV-	GSE182227	6911	Primary
OP13	Male	HPV+	GSE182227	744	Primary
OP14	Male	HPV+	GSE182227	3238	Primary
OP16	Male	HPV-	GSE182227	3330	Primary
OP17	Male	HPV+	GSE182227	1163	Primary
OP19	Female	HPV-	GSE182227	2274	Primary
OP20	Male	HPV+	GSE182227	6187	Primary
OP33	Male	HPV+	GSE182227	7212	Primary
OP34	Male	HPV+	GSE182227	7232	Primary
OP35	Male	HPV+	GSE182227	1167	Primary
OP4	Male	HPV+	GSE182227	5006	Primary
OP5	Male	HPV+	GSE182227	2664	Primary
OP6	Female	HPV+	GSE182227	3269	Primary
OP8	Male	HPV-	GSE182227	6964	Primary
OP9	Male	HPV+	GSE182227	6531	Primary
P15	Female	HPV-	GSE181919	1731	Primary
P21	Male	HPV-	GSE181919	697	Primary
P22	Male	HPV+	GSE181919	1143	Primary
P26	Male	HPV-	GSE181919	944	Primary
P30	Male	HPV-	GSE181919	282	Primary
P31	Male	HPV-	GSE181919	523	Primary
P38	Male	HPV-	GSE181919	1049	Primary
P4	Female	HPV-	GSE181919	608	Primary
P43	Male	HPV+	GSE181919	1662	Primary
P46	Male	HPV+	GSE181919	1413	Primary
P51	Male	HPV-	GSE181919	1801	Primary
P57	Male	HPV+	GSE181919	1104	Primary
P59	Male	HPV+	GSE181919	2350	Primary
P6	Male	HPV-	GSE181919	1327	Primary
P60	Female	HPV-	GSE181919	1948	Primary
P7	Male	HPV-	GSE181919	536	Primary
P8	Male	HPV-	GSE181919	1206	Primary
P84	Male	HPV+	GSE181919	830	Primary
P86	Female	HPV+	GSE181919	1084	Primary
P9	Female	HPV-	GSE181919	850	Primary

Supplementary Table 3. Proportion of each major cell type across female and male HNSCC patient groups, separated by HPV status (HPV+ and HPV−).

	Percentage All Cell Types			
	HPV -		HPV +	
	Female	Male	Female	Male
Bcell	4.4%	3.6%	7.7%	10.20%
CD4T	23.9%	18.7%	22.5%	20.00%
CD8T	21.7%	17.2%	9.5%	16.30%
Endothelial	8.9%	6.8%	17.0%	4.90%
Epithelial	1.6%	2.0%	1.6%	2.00%
Fibroblast	6.7%	10.9%	18.3%	5.50%
Macrophage	4.2%	5.9%	1.3%	2.90%
Mast	0.5%	0.9%	1.7%	0.40%
Monocyte	7.8%	4.2%	0.8%	0.80%
Multi	1.2%	0.9%	1.4%	0.90%
NK	5.5%	3.1%	1.0%	2.70%
Neutrophils	1.6%	2.2%	0.0%	0.00%
PlasmaCell	0.9%	3.0%	3.5%	4.60%
Tumor	8.4%	16.9%	11.2%	26.10%
panDC	2.9%	3.7%	2.7%	2.90%

Supplementary Table 4. Proportion of each major immune cell type across female and male HNSCC patient groups, separated by HPV status.

	Percentage Immune Cell Types			
	HPV -		HPV +	
	Female	Male	Female	Male
Bcell	6.0%	6.0%	16.3%	18.2%
CD4T	33.0%	31.4%	47.7%	35.6%
CD8T	29.9%	28.8%	20.1%	29.0%
Macrophage	5.9%	9.9%	2.8%	5.2%
Mast	0.7%	1.5%	3.7%	0.7%
Monocyte	10.7%	7.1%	1.6%	1.4%
NK	7.5%	5.2%	2.1%	4.8%
Neutrophils	2.3%	3.8%	0.0%	0.1%
panDC	4.0%	6.3%	5.7%	5.1%

Supplementary Table 5. Proportion of CD8+ T cell subtypes across female and male HNSCC patient groups, separated by HPV status.

Percentage CD8 Cell Types				
	HPV -		HPV +	
	Female	Male	Female	Male
CD8.CM	23.0%	14.8%	37.1%	20.3%
CD8.EM	29.6%	35.4%	28.5%	40.2%
CD8.MAIT	0.5%	0.4%	0.0%	0.4%
CD8.NaiveLike	0.8%	1.0%	1.3%	2.2%
CD8.TEMRA	1.2%	1.3%	0.3%	0.4%
CD8.TEX	43.3%	45.5%	26.6%	33.6%
CD8.TPEX	1.6%	1.6%	6.2%	3.0%

Supplementary Table 6. Proportion of CD4+ T cell subtypes across female and male HNSCC patient groups, separated by HPV status.

Percentage CD4 Cell Types				
	HPV -		HPV +	
	Female	Male	Female	Male
CD4.CTL_EOMES	1.2%	2.7%	12.8%	2.9%
CD4.CTL_Exh	8.0%	6.0%	1.2%	3.5%
CD4.CTL_GNLY	1.5%	2.0%	12.5%	2.1%
CD4.NaiveLike	29.5%	33.8%	46.3%	47.9%
CD4.Tfh	12.4%	9.3%	11.2%	11.5%
CD4.Th17	2.0%	3.9%	2.8%	3.2%
CD4.Treg	45.5%	42.2%	13.1%	28.9%

Supplementary Table 7. Proportion of dendritic cell (DC) subtypes across female and male HNSCC patient groups, separated by HPV status.

Percentage DC Cell Types	HPV -		HPV +	
	Female	Male	Female	Male
AS-DC	2.3%	5.0%	1.0%	1.5%
DC3	23.1%	20.5%	29.5%	18.9%
MonoDC	1.7%	0.9%	1.0%	2.4%
cDC1	9.2%	8.4%	17.1%	12.1%
cDC2_CD1A	5.4%	5.7%	6.7%	10.1%
cDC2_CLEC10A	27.4%	34.6%	24.8%	28.1%
pDC	30.9%	25.0%	20.0%	27.0%

Supplementary Table 8. Overview of CD8⁺, CD4⁺, and dendritic cell subtypes.

This table provides an overview of major CD8⁺ T cell, CD4⁺ T cell, and dendritic cell (DC) subtypes, including their definitions and functional roles in immune regulation. This table serves as a reference for the classification and functional annotation of immune cell subsets in HNSCC tumors, facilitating comparisons across different studies.

Cell Type	Description
CD8.NaiveLike	Antigen-naive T cells
CD8.CM	Central Memory T cells
CD8.EM	Effector Memory T cells
CD8.TEMRA	Effector Memory cells re-expressing CD45RA. Sometimes called Short Lived Effectors (SLEC), or Cytotoxic effectors
CD8.TPEX	Progenitor exhausted T cells
CD8.TEX	Exhausted T cells
CD8.MAIT	Mucosal-associated invariant T cells, innate-like T cells defined by their semi-invariant αβ T cell receptor
CD4.NaiveLike	T cells with naive-like phenotype
CD4.Tfh	T follicular helper cells
CD4.Th17	Th17 helper cells
CD4.Treg	T regulatory cells
CD4.CTL_EOMES	Cytotoxic CD4 T cells expressing EOMES and GZMK
CD4.CTL_GNLY	Cytotoxic CD4 T cells expressing GNLY
CD4.CTL_Exh	Cytotoxic CD4 T cells with exhaustion phenotype
AS-DC	AXL+ SIGLEC6+ Dendritic Cells, also referred to as DC5 or Pre-DCs
cDC1	Conventional Dendritic Cells type 1, specialized in antigen cross-presentation and CD8+ T cell activation
cDC2_CD1A	Conventional Dendritic Cells type 2 subset expressing CD1A, involved in CD4+ T cell activation
cDC2_CLEC10A	Conventional Dendritic Cells type 2 subset expressing CLEC10A, functionally distinct from CD1A-expressing cDC2
DC3	Tissue-resident DCs, lacking a direct counterpart in circulation, potentially derived from cDC1 or cDC2/MoDC
MonoDC	Monocyte-derived Dendritic Cells, inflammation-induced, with similarities to cDC2
pDC	Plasmacytoid Dendritic Cells, key producers of type I IFNs, fostering antitumor immunity
